# Advances in bladder substitution and creation of neobladders in children

**DOI:** 10.12688/f1000research.16607.1

**Published:** 2019-11-26

**Authors:** Jennifer E. Reifsnyder, Moneer K. Hanna

**Affiliations:** 1 Division of Pediatric Urology, Cohen Children's Medical Center, Northwell Health, New Hyde Park, NY, 11040, USA; 2 Division of Pediatric Urology, New York Presbyterian Hospital-Weill Cornell Medicine, New York, NY, 10065, USA

**Keywords:** cystectomy, urinary bladder, urinary diversion, continent urinary reservoirs, pediatrics

## Abstract

Cystectomy and urinary bladder substitution are rare in children but may be necessary in some cases of bladder exstrophy, in the setting of malignancy, or in other settings in which the bladder is severely dysfunctional. This article details advances in surgical techniques in creating continent urinary diversions in this specialized pediatric population.

Urinary bladder substitution is an uncommon surgical procedure in children because every effort is made to preserve the native bladder. Frequently, medical treatment and surgical procedures to augment the bladder or to enhance its competence, or sometimes to close the bladder outlet and create a catheterizable channel, are performed in order to preserve the bladder regardless of bladder size. However, in select patients, bladder augmentation is impossible and bladder substitution is required. This occurs in several scenarios. In some cases of malignancy, such as following treatment of genitourinary rhabdomyosarcoma with a combination of chemotherapy and radiotherapy, the bladder becomes fibrotic and functionally compromised. These children are candidates for cystectomy and bladder substitution with either a neobladder or a continent catheterizable diversion. Other select cases include patients with bladder exstrophy and small bladder plates with polypoid changes that have remained exposed for prolonged periods of time, which may undergo malignant changes. This occurs particularly in underdeveloped countries where treatment and follow-up are challenging. Indeed, this has been documented in 10% of cystectomy specimens in this specialized population ^1^. Ko *et al*. ^2^ reported 18 patients with classic bladder exstrophy, bladder exstrophy variants, and cloacal exstrophy who required cystectomy because their bladder templates were of insufficient size and the compliance and tissue quality did not improve after bladder closure. Six out of 18 patients underwent continent cutaneous diversion at the time of cystectomy and two more patients underwent delayed continent cutaneous diversion; all were continent on intermittent catheterization. Some dysfunctional bladders without drainage become a closed system, especially after urinary diversion, and become a source of recurrent sepsis in the pelvis, such as occurs in some cases of bladder exstrophy with epispadias in the presence of retrograde flow of prostatic and ejaculate fluid. There have been multiple advances in the surgical techniques used to treat the complex problem of bladder substitution in these varied populations. Neobladder creation permits an orthotopic urinary reservoir and the potential to empty the reservoir either through external sphincter relaxation with Valsalva voiding or via intermittent catheterization, whereas continent cutaneous urinary diversions offer a continent solution for patients for whom urinary continence with an orthotopic neobladder is not possible.

Bladder substitution may be performed by using either the small bowel or the large bowel. In either case, the principle of detubularization remains unchallenged. In addition to causing the “dyscoordination” of the peristaltic contractions, the conversion of a tubular segment of intestine into a spherical reservoir of bowel decreases the pressure and peristalsis and increases the capacity of the segment. For example, a 20 cm segment of bowel with a diameter of 3.4 cm has a volume of 158 cm. When detubularized and folded on itself to become 10 cm long with a diameter of 6.8 cm, the volume increases to 316 mL. A detubularized segment also allows for a more spherical configuration, resulting in a lower surface area relative to the volume of the reservoir, thus minimizing the likelihood of metabolic complications.

The authors prefer the creation of a urinary reservoir using the terminal ileum, cecum, and ascending colon based on the right and ileocolic vessels. The bowel is opened on the anti-mesenteric border and reconfigured as a U-shaped reservoir and then the ureters are reimplanted in the cecal wall and the pouch is closed. The appendix remains unchanged on the cecum and is positioned so that it may be used as an orthotopic neourethra or as an ectopic umbilical stoma, depending on the type of reconstruction ^3^. Intraoperatively, the cecal appendiceal junction is assessed for competence prior to completion of the cecal closure. A cystostomy tube is inserted in the cecal part of the bladder, and the tip of the appendix is removed. Then normal saline is run through the tube until the bladder is filled. The saline is then infused by gravity at 60 cm above the bladder level to test the competence of the cecal appendiceal sphincter and to ensure a watertight anastomosis. Subsequently the cecum is plicated over the base of the appendix to reinforce the sphincteric mechanism.

The importance of preventing reflux of urine from a urinary reservoir created from bowel into the upper urinary tract has not been demonstrated conclusively in the adult population but remains unchallenged in the pediatric population, where the urinary diversion will be required to carry the patient through childhood and into and through adulthood. Reflux prevention is particularly important when there is a marked pressure gradient between the upper tract and the cystoplasty reservoir as a result of either the peristaltic activity of an unaltered bowel segment or a high-pressure detubularized bowel segment. Reflux naturally predisposes to ascent of infection from a substituted bladder to the patient’s upper urinary tract. The principles of tunnel or nipple valves are used for reflux prevention of urethral intestinal implantation and are similar to those used for vesico-ureteric reimplantation. Structural differences in the bowel wall may involve some procedural variations. However, the principles of the procedures are technically similar in that filling of the reservoir compresses the ureteral lumen, thus preventing reflux of urine. In the tunnel procedures, the lumen lies obliquely, so that the ureter is laterally compressed against the wall of the reservoir. A ureteral diameter of 2 to 3 mm (6 to 9 French) and a 2 to 3 cm tunnel create a ratio of 8 to 1. Alternatively, in the cuff-nipple procedure, the lumen is concentric and is closed by a circumferential rise in pressure. The critical dimensions are that the nipple length is twice the center diameter. However, the disadvantages of the nipple procedure are that compression of the outer layer results in strangulation of the inner valve and that pulling outwards and retraction and eversion result in partial inefficiency of this valve mechanism. However, suturing the posterior wall of the split cuff-nipple to the denuded mucosal wall of the bladder eliminates the possibility of eversion of the valve and therefore increases the efficiency of the nipple valve mechanism.

Abol-Enein and Ghoneim ^4^ described an ingenious inter-serosal space procedure for the implantation of the ureters into a detubularized bowel segment reservoir which involves simply sandwiching them between the layers of tubularizing folds in order to create a serous-lined extramural tunnel. This creates a reflux-preventing valve that is compressed by intraluminal pressure. The advantages of this procedure are its simplicity and the fact that it is suitable for ureters of normal size as well as those that are grossly dilated and thick-walled.

In addition to neobladder creation, bladder substitution may be accomplished with continent cutaneous diversion in the pediatric population as well. The Kock pouch, originally used for continent ileostomy creation ^5^, has made a major contribution to continent urinary diversion when adapted for use in the urinary tract and has become a desirable alternative to the standard ileal conduit. Short- and long-term follow-up were associated with significant complications because of malfunction of the nipple valve. Various operative technical modifications, including use of a Dacron fabric collar instead of Marlex mesh and anchoring the nipple to the wall of the reservoir, were developed to improve the results. However, long-term results of the Kock pouch in children remain unknown. Further adaptation of the Kock pouch in children has resulted in its use as an orthotopic bladder substitute as well ^6, 7^.

The T-Pouch ^8^ was originally reported as a surgical procedure using the ileum for continent diversion following radical cystectomy for bladder cancer. The procedure combines the use of the serous-lined valve previously discussed and the tapered bowel Monti procedure ^9^. The T-pouch is another ingenious procedure which lends itself to use in children; indeed, we have used it in three cases and have been impressed by its simplicity and versatility. In children, the T-pouch involves isolation of a segment of small bowel (30–40 cm), which then is subdivided into two segments. The first section of bowel (20–25 cm) is detubularized to form the reservoir (350–450 mL capacity after closure) and then a long incision (7–10 cm) in the mesentery is made between the first and second segments. The purpose of the mesenteric incision is to allow for free mobilization of the afferent limb of the valve. The second segment, which will form the afferent limb, is tapered over a 12-French catheter and sutured in two layers and then implanted in the serous-lined tunnel of the reservoir to create a continent catheterizable channel. The ureters are implanted in the reservoir by using the split cuff-nipple method.

In the adult bladder cancer population, the Studer pouch has been widely used and was the focus of a randomized clinical trial (USC-STAR) comparing renal function three years after creation of either a Studer pouch or a T-pouch in adult patients with bladder cancer ^10^. The Studer pouch uses a long segment of detubularized ileum for pouch creation as well as a shorter segment of intact ileum as an afferent limb, into which the ureters are implanted in a non-refluxing fashion. The trial found no difference in renal function between the two groups and concluded that, in this population, the anti-refluxing mechanism was not advantageous ^10^. Given the much longer expected life span of pediatric patients and therefore prolonged duration of anticipated exposure to reflux into the renal units of pediatric patients undergoing urinary diversion, the Studer pouch has not gained favor in this population.

The I-pouch has been described in adults and offers a potential alternative method for neobladder creation, but so far the results of this technique have not been reported in a pediatric population ^11, 12^. In this technique in adults, a 40 cm ileal segment is isolated about 20 cm proximal to the ileocecal valve and is folded into a U shape such that each limb has a length of 20 cm and the open ends are adjacent to one another. Close to the open ends of the ileum, the adjacent paramesenteric borders of each ileal limb are sutured together for about 8 cm. The ileal loops are then opened along the anti-mesenteric borders. However, this is carried out in an asymmetrical fashion to create a narrower center section of detubularized ileum adjacent to the sutured paramesenteric borders of each limb (about 8 cm). Toward what will become the center of the pouch, this cut is taken inward toward the mesentery for a short distance and then sharply outward to create a symmetric detubularization at the U-turn in the ileum. The narrower section of the detubularized ileum between the ileal limbs is used to create a serous-lined valve into which the ureters are implanted by using a modified Wallace technique. The remainder of the ileum is folded on itself and sutured together in spherical fashion, and a urethral opening is created at the dependent portion of the pouch. This technique is difficult to use if a neourethra is needed, but it does provide the distinct advantage of ease in catheterizing the upper tracts at a later time, if necessary.

In some patients, intermittent catheterization, whether via a continent cutaneous urinary diversion or an orthotopic neobladder, is impractical or unacceptable for social reasons or because of a lack of resources. The Mainz II internal diversion ^13^ offers a practical solution and is a reasonable option, especially for females and for those who have endured multiple reconstructive surgical failures. The original technique of detubularizing the sigmoid colon by making an inverted U-shaped incision has been modified by instead making an inverted W-shaped incision primarily in order to allow for implantation of the two ureters into extramural tunnels and additionally to increase the capacity of the sigmoid reservoir.

Various authors have chronicled outcomes among pediatric populations with urinary bladder substitution or neobladder. Stein *et al*. described outcomes of several types of urinary diversion in 19 patients with bladder or prostate rhabdomyosarcoma ^14^. Urinary diversion included incontinent diversion in four patients and the remainder underwent continent cutaneous ileocecal pouch/Mainz I pouch (11 patients), urethral ileocecal pouch/orthotopic Mainz I pouch (two), ureterosigmoidostomy (one), or Mainz II pouch (two). All surviving patients were continent, and those with an orthotopic neobladder were continent and voided spontaneously. Eight out of 14 survivors required operative intervention for complications varying from pouch calculi to fistulae; this cohort was followed for a median of 132 months, and some complications occurred as long as 22 years after initial urinary diversion creation.

In 2016, Deuker *et al*. described outcomes of Mainz pouch or ileocecal pouch in 107 children, and the median follow-up was nearly 16 years ^15^. The population included patients with neurogenic bladder, exstrophy-epispadias complex, and malignancy. Stomal stenosis was a significant problem, affecting 38% of patients with an appendiceal stoma and 20% of patients with an intussuscepted ileal nipple valve. 89% of patients with an appendiceal stoma and 82% of patients with an intussuscepted ileal nipple valve were continent. The majority (99%) of patients surveyed were satisfied with the type of bladder substitution received.

Chowdhary *et al*. detailed results of the Indiana pouch in a pediatric population that included patients with exstrophy-epispadias complex and multiple failed repairs, cloacal exstrophy, rhabdomyoscarcoma, and neuropathic bladder with diffuse neoplastic polyposis ^16^. The authors reported on 12 patients with a median follow-up of 2 years (1 to 15 years), and all patients endorsed dry intervals of 4 hours or longer. Complications included stomal stenosis requiring revision in one patient and wound dehiscence in another.

Several groups have reported favorable continence outcomes of the Mainz pouch II. Mingin *et al*. ^17^ described five patients with bladder exstrophy who underwent the Mainz pouch II procedure between 1996 and 1998 to create a rectosigmoid pouch, allowing urine to drain into and be eliminated via the rectum. Three patients required oral sodium bicarbonate to correct a metabolic acidosis, but upper tracts remained non-dilated, all patients were continent during daytime and nighttime, and there were no episodes of pyelonephritis during follow-up, which was between about 1 and 3 years.

Rubenwolf *et al*. ^18^ recently reported long-term follow-up after continent anal diversion and described a cohort of 82 exstrophy patients who underwent continent anal urinary diversion between 1970 and 2015 (57 were eligible and 32 of them chose to participate). The median age was 38.6 years. One patient reported daytime incontinence, and 11 patients reported occasional small-volume nighttime incontinence and six (all female) of these 11 required a pad at night. Three female patients reported small-volume urine loss during intercourse. Eight had recurrent pyelonephritis, two had undergone nephrectomy for non-functioning kidney, and one patient was converted to an ileal conduit because of loss of renal function. The group reported that 20% of upper tract complications occurred after a Mainz pouch II procedure and the other 80% of complications occurred after traditional ureterosigmoidostomy.

There are several significant long-term consequences of introducing bowel into the urinary tract, which lead to recognition that creation of a bladder substitute using bowel segments is in many ways merely a substitution of an immediate issue for a long-term issue. In a comprehensive review of the metabolic consequences and long-term complications of incorporating bowel in the urinary tract, Gilbert and Hensle concluded that metabolic acidosis is the most common metabolic abnormality, which can lead to loss of bone mineral density and development of osteoporosis with increased risk of pathologic fractures ^19^. Furthermore, the authors discussed the morbidity of the loss of various portions of gastrointestinal tract, which can interfere with its normal function.

When selecting a method of urinary diversion there is concern for higher risk of later malignancy which must be taken into account. In Rubenwolf *et al*., eight out of 82 patients (9.8%) developed cancer following continent anal diversion, and the diagnosis of the cancer ranged from 15 to 48 years at a median of 34 years after creation of the diversion ^18^. Ragu *et al*. ^20^ evaluated the risk of carcinoma in patients with ureterosigmoid anastomoses in a systematic review. Twenty-three patients who had undergone ureterosigmoidostomy or rectal neobladder and subsequently developed carcinoma (22 adenocarcinoma and one carcinoid tumor) were identified. In 26.1% of patients with diagnosed cancer, the diagnosis was obtained only during surveillance. In half of the remaining patients who did not undergo surveillance endoscopy, advanced cancer or obstructive symptoms were diagnosed in an emergency setting. The median age at urinary diversion was 3 years, and the median time from surgery for urinary diversion to diagnosis of cancer was 31 years (range of 5 to 55 years).

## Minimally invasive techniques for urinary diversion

Minimally invasive techniques, specifically robot-assisted laparoscopic techniques, have transformed the way radical cystectomy with urinary diversion is performed for adult patients with bladder cancer over the past 15 years. Several randomized controlled clinical trials have evaluated the complication profile and oncologic outcomes of open versus robot-assisted laparoscopic techniques and found the robot-assisted techniques to be of comparable efficacy; however, the urinary diversion was typically performed in an open fashion in these trials ^21– 24^. More recently, robot-assisted intracorporeal urinary diversion has become an increasingly accepted technique for adult patients undergoing radical cystectomy and has a similar and possibly improved complication profile ^25– 27^. In addition, in a porcine model evaluating perioperative inflammatory marker levels at the time of radical cystectomy and open versus robot-assisted urinary diversion, inflammatory markers were similar between the intracorporeal diversion groups and mini-laparotomy group ^28^. Currently, two open randomized trials—one in the UK (ClinicalTrials.gov Identifier: NCT03049410) ^29^ and another in Italy (ClinicalTrials.gov Identifier: NCT03434132)—plan to compare outcomes for bladder cancer patients undergoing radical cystectomy with either intracorporeal or open urinary diversion.

It is possible that the robotic platform will prove useful for select pediatric patients as well. As minimally invasive techniques become more widely used, pediatric urologists have adopted them for many other applications, including complex procedures such as augmentation cystoplasty and appendicovesicostomy ^30^ and even revision appendicovesicostomy in a patient with a previously created neobladder ^31^. However, in pediatric patients, extra caution should be exercised, and care should be individualized on the basis of the indication for cystectomy and neobladder, as minimally invasive techniques may not be advisable for all pediatric patients.

## Future options

The use of bowel in the urinary tract has allowed the creation of a continent bladder substitution, and a catheterizable channel if needed, in children who otherwise would be unable to achieve urinary continence. However, multiple mechanical and metabolic complications arise from usage of bowel. Ideally, an inert material would be used to create a tissue-engineered bladder to replace bowel in order to avoid these complications.

Phase I and II trials have been conducted for patients with spina bifida to evaluate the safety and efficacy of an autologous tissue-engineered bladder patch in place of traditional augmentation with a segment of bowel. Urothelial and smooth muscle cells from each enrolled patient’s own cells were grown in culture for 7 weeks and then augmentation cystoplasty was performed by using the autologous bladder patch. The phase I trial showed encouraging results ^32^, but the phase II trial was disappointing because of the inability to demonstrate improvement in bladder capacity or compliance in addition to the high complication rate requiring surgical intervention ^33^.

Two clinical trials—“Incontinent Urinary Diversion Using an Autologous Neo-Urinary Conduit” (ClinicalTrials.gov Identifier: NCTO1087697, phase I trial) and “Study of Autologous Neo-Bladder Construct in Subjects With Neurogenic Bladder Following Spinal Cord Injury” (ClinicalTrials.gov Identifier: NCTOO512148, phase II trial)—have completed enrollment using engineered autologous bladder tissue to create urinary conduits or reservoirs for other applications. Though indirectly related to children who need bladder substitution, this research offers the possibility of leading to other options for these children in the future.
